# N‐terminomics and proteomics analysis of Calpain‐2 reveal key proteolytic processing of metabolic and cell adhesion proteins

**DOI:** 10.1002/pro.70144

**Published:** 2025-04-25

**Authors:** Anjali Kapilan, Mitchell Bulluss, Alexander R. Ziegler, Mohamed Dabaja, Afshin Derakhshani, Anthonia Anowai, Victoria Armstrong, Rhiannon Campden, Daniel Young, Young Joo Sun, Nichollas E. Scott, Laura E. Edgington‐Mitchell, Vinit B. Mahajan, Antoine Dufour

**Affiliations:** ^1^ Department of Physiology & Pharmacology University of Calgary Calgary Alberta Canada; ^2^ Department of Biochemistry and Molecular Biology University of Calgary Calgary Alberta Canada; ^3^ McCaig Institute for Bone and Joint Health University of Calgary Calgary Alberta Canada; ^4^ Snyder Institute for Chronic Diseases University of Calgary Calgary Alberta Canada; ^5^ Hotchkiss Brain Institute University of Calgary Calgary Alberta Canada; ^6^ Department of Biochemistry and Pharmacology, Bio21 Molecular Science and Biotechnology Institute The University of Melbourne Parkville Victoria Australia; ^7^ Molecular Surgery Laboratory, Department of Ophthalmology, Byers Eye Institute Stanford University Palo Alto California USA; ^8^ Department of Microbiology and Immunology Peter Doherty Institute, The University of Melbourne Parkville Victoria Australia; ^9^ Veterans Affairs Palo Alto Health Care System Palo Alto California USA

**Keywords:** Calpain‐2, metabolomics, N‐terminomics, protease, proteomics, THP‐1 monocytes

## Abstract

Aberrant levels of the cysteine protease Calpain‐2 have been linked to neurodegeneration, inflammation, and cancer, yet our understanding of this protease and its substrates remains limited. Systematic studies to identify Calpain‐2 substrates have been largely confined to peptide libraries or in vitro studies, which fail to represent physiological cellular conditions and physiologically relevant substrates. To identify existing and novel Calpain‐2 substrates, we used a genetic approach to knockout Calpain‐2 in the THP‐1 human monocyte‐like cells, followed by proteomic and N‐terminomic/TAILS mass spectrometry approaches to identify Calpain‐2 substrates. We identified 51 substrates that may be cleaved directly by Calpain‐2 or indirectly by downstream proteases. The direct cleavage of selected substrates by Calpain‐2 was confirmed using in vitro assays. Finally, metabolomics analysis identified a role for Calpain‐2 in the regulation of pyrimidine and glutathione metabolism. Our unbiased and quantitative mass spectrometry analytical pipeline provides new evidence on the physiological functions of Calpain‐2 and its newly identified substrates in THP‐1 cells.

## 1 INTRODUCTION

Calpains are cytosolic cysteine proteases that function at neutral pH (Ono et al. 2016; Vu et al. 2022; Zatz and Starling 2005). Calpain‐1 and Calpain‐2 are ubiquitous and have similar heterodimer structures requiring interaction with the calpain small subunit 1 (CAPNS1) and Ca^2+^ for activity (Ono et al. 2016). Calpain‐1 requires micromolar concentrations of calcium, while Calpain‐2 requires millimolar amounts of calcium (Ono et al. 2016). Calpastatin (CAST) is a high‐affinity endogenous inhibitor of Calpains that regulates their functions (Nian and Ma 2021). Calpain‐1 and ‐2 depend on calcium to initiate catalysis, and importantly, there is typically no quantifiable proteolytic activity in the absence of calcium (Goll et al. 2003). Crystallography and calcium‐activation studies demonstrated that calcium binding to the 29‐residue EF‐hand domains, which consist of an α‐helix “E” that may bind calcium with a second α‐helix “F” triggers a conformational change, allowing key histidine, cysteine, and asparagine residues to form a close‐knit catalytic triad (Strobl et al. 2000).

Identification of proteins cleaved by Calpains and deciphering the consequences of the cleavage is essential to fully understanding their biological functions. Calpain substrates are suspected to be extensive as there is no known preferred signature, consensus target sequence, or conformation that Calpains favor. This is in contrast to protease systems such as trypsin, caspases, and ubiquitin‐proteasomes that have selectivity for specific amino acids (Klein et al. 2018; Ono et al. 2016; Sorimachi and Ono 2012; Vu et al. 2022; Wang et al. 2021). Calpain substrates have been identified using targeted approaches in various cell types and cellular contexts involving elevated intracellular Ca^2+^ (e.g., cytoskeletal remodeling, apoptosis, signal transduction, and transcription regulation); however, there are likely many more that have yet to be identified (Goll et al. 2003; Ono et al. 2016; Pontremoli et al. 1990; Sorimachi and Ono 2012; Storr et al. 2011). Based on the bioinformatic tool TopFIND (Fortelny et al. 2015; Lange and Overall 2011), known substrates for Calpain‐2, identified in various cell types but mainly neuronal cells, include Integrin beta‐2 (Pfaff et al. 1999). Nuclear factor of kappa light polypeptide gene enhancer in B‐cells inhibitor alpha (IκBα) (Han et al. 1999), Cyclin‐dependent kinase‐5 (Cdk5) (Patzke and Tsai 2002), Caspase‐8 (Chua et al. 2000), Caspase‐9 (Chua et al. 2000), and many more substrates linked to neuronal functions such as dopamine transport (Franekova et al. 2008) or myelin basic protein (MBP) (Banik et al. 1994). However, most of these substrates have not been validated in cells or in vivo. It is also not fully understood whether these substrates are unique to Calpain‐2 or if other Calpains can also cleave these proteins via compensatory mechanisms. One challenge in finding evidence for distinct Calpain‐1 and Calpain‐2 substrates is the lack of a selective inhibitor for either protease. Most, if not all, Calpain inhibitors have poor selectivity for individual Calpains, thus making it challenging to characterize the unique roles of Calpain. Additionally, many Calpain inhibitors also inhibit other cysteine proteases such as cathepsins or legumain and are often not properly reported in the literature. Therefore, our approach to identifying the unique repertoire of Calpain‐2 substrates involves unbiased quantitative proteomics and N‐terminomics. N‐terminomics/TAILS (Agbani et al. 2023; Das et al. 2021; Das et al. 2023; Kleifeld et al. 2010; Wang et al. 2021) is an ideal technique to profile both the N‐terminome and proteome regulated and impacted by a protease. TAILS has been used in various tissues (Anderson et al. 2020; Bellac et al. 2014; Das et al. 2023; de Almeida et al. 2022a; Gordon et al. 2019; Mainoli et al. 2020; Ziegler et al. 2024), serum (Agbani et al. 2023), synovial fluid (Das et al. 2023), and various cells (Klein et al. 2015; Marchant et al. 2014). We selected THP‐1 human monocyte‐like cells, which are an immortalized cell line derived from an acute monocytic leukemia patient and are commonly employed to model human peripheral blood monocytes. While Calpain‐1 and Calpain‐2 share some overlapping substrates, we identified distinct substrates specific to Calpain‐2. We identified the known Calpain‐2 substrates along with over 51 novel putative Calpain‐2‐mediated cleavage events in THP‐1 cells, which likely include direct and indirect substrates. This work highlights key potential biological functions of Calpain‐2, which will permit future distinction from the roles of Calpain‐1.

## 2 RESULTS

### 2.1 Reduction of antigen presentation capacity and proinflammatory activation in PMA‐treated Calpain‐2‐deficient macrophages

With the aim of understanding the role of Calpain‐2 in macrophages, we generated THP‐1 *CAPN2* ^*−/−*^ cells using CRISPR‐Cas9 gene editing. To avoid the limited lifespan and heterogeneity of monocyte‐derived human macrophages, we used THP‐1 cells derived from a patient with acute monocytic leukemia (Chanput et al. 2014; Tedesco et al. 2018). We used Western blotting analysis to verify that the expression of Calpain‐2 protein in *CAPN2* ^*−/−*^ THP‐1 cells was negligible (Figure S1a, Supporting Information). Using a proliferation assay, we observed a reduction in the number of *CAPN2* ^*−/−*^ THP‐1 cells over time compared to WT cells, which may indicate that the loss of Calpain‐2 either impairs cell proliferation or promotes cell death (Figure S1b). In support of the latter, to avoid confounding factors associated with this difference, we used equal numbers of early passage cells for all experiments.

**FIGURE 1:**
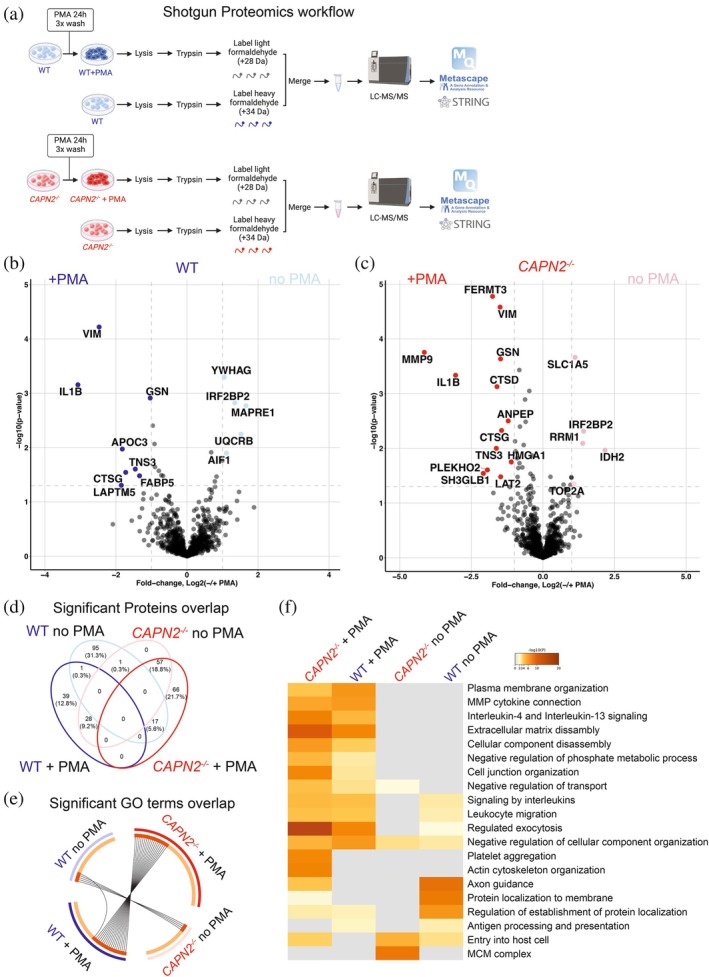
Proteomics analysis of human WT and *CAPN2* ^*−/−*^ THP‐1 cells untreated or treated with PMA. (a) Workflow schematic of proteomics experimental design. THP‐1 were untreated or treated with phorbol 12‐myristate 13‐acetate (PMA) for 24 h to generate macrophage‐like cells (*n* = 4 for each group). Note that in this experiment, Calpain‐2 was not activated. Proteins were digested with trypsin, labeled with either light or heavy formaldehyde and subjected to LC–MS/MS and analyzed using MaxQuant at 1% FDR. Graphic created using BioRender. (b) Proteins from WT cells were analyzed by a two‐way *t* test and visualized by volcano plot where significance is defined as intensities (log2(−/+PMA)) > 1 and −log10(p) > 1.3. (c) Proteins from *CAPN2* ^*−/−*^ cells were analyzed by a two‐way *t* test and visualized by volcano plot where significance is defined as intensities (log2(−/+PMA)) > 1 and −log10(p) > 1.3. (d) Venn diagram of the statistically changing proteins among the four groups (>1.5 fold and <0.66 using an interquartile boxplot analysis; Spitzer et al. 2014). The complete list of proteins identified is shown in Tables S1 and S2. (e) Circos plot indicating the overlapping Gene Ontology (GO) terms between WT and *CAPN2* ^*−/−*^ THP‐1 cells untreated or treated with PMA. Data analyzed from four biological replicates. (f) Metascape (Zhou et al. 2019) analysis of different pathways between WT and *CAPN2* ^*−/−*^ THP‐1 cells untreated or treated with PMA. Accumulative hypergeometric p‐values and enrichment factors were calculated and used for filtering. Remaining significant terms were then hierarchically clustered into a tree based on Kappa‐statistical similarities among their gene's memberships. Then, 0.3 kappa score was applied as the threshold to cast the tree into term clusters.

Treatment of THP‐1 cells with phorbol 12‐myristate 13‐acetate (PMA) stimulates their differentiation to a macrophage‐like phenotype (Lund et al. 2016; Tedesco et al. 2018). To investigate whether Calpain‐2 regulates or is regulated by other proteases as part of the protease web (Fortelny et al. 2014), we first aimed to investigate if the levels of Calpain‐2 or other proteases were impacted by PMA activation of THP‐1 cells. Using quantitative proteomics, we analyzed the protein abundance differences between WT and *CAPN2* ^*−/−*^ THP‐1 cells with and without PMA activation (Figure 1a). Using cutoffs of >1.5 fold and below <0.66 fold, we identified 68 proteins elevated in WT THP‐1 cells after PMA activation and 112 proteins elevated in WT THP‐1 cells before PMA activation (Figure 1b–d and Table S1). Among these, there were seven and five high‐confidence hits, respectively, that were identified in at least three of four replicates with a *p* value <0.05 (Figure 1b,c). We identified 86 proteins elevated in *CAPN2* ^*−/−*^ THP‐1 cells after PMA activation and 81 proteins elevated in *CAPN2* ^*−/−*^ THP‐1 cells before PMA activation, including 15 and 5 high‐confidence hits, respectively (Figure 1c and Table S2). We detected no compensatory response by any other Calpains or the endogenous Calpain inhibitor Calpastatin in response to Calpain‐2. Using Metascape (Zhou et al. 2019) analysis, we generated an overlap circos plot, and we identified increased overlap between WT and *CAPN2* ^*−/−*^ THP‐1 cells after PMA activation (Figure 1e,f). These results suggest that PMA activation drives more significant proteome changes than the genetic knockout of Calpain‐2. Of note, Vimentin (VIM), Interleukin‐1β (IL1B), Gelsolin (GSN), Cathepsin G (CTSG), and Tensin‐3 (TNS3) were significantly increased by PMA in both genotypes, while IRF2BP2 was downregulated in both genotypes. Using Metascape (Zhou et al. 2019) analysis, we generated a heatmap of Gene Ontology (GO) terms (Figure 1d). Interestingly, *CAPN2* ^*−/−*^ THP‐1 cells lack an enrichment of antigen processing and presentation as compared to WT THP‐1 cells, suggesting a defect in macrophage function during antigen presentation.

### 2.2 N‐terminomics/TAILS analysis revealed potential novel substrates of Calpain‐2

**FIGURE 2:**
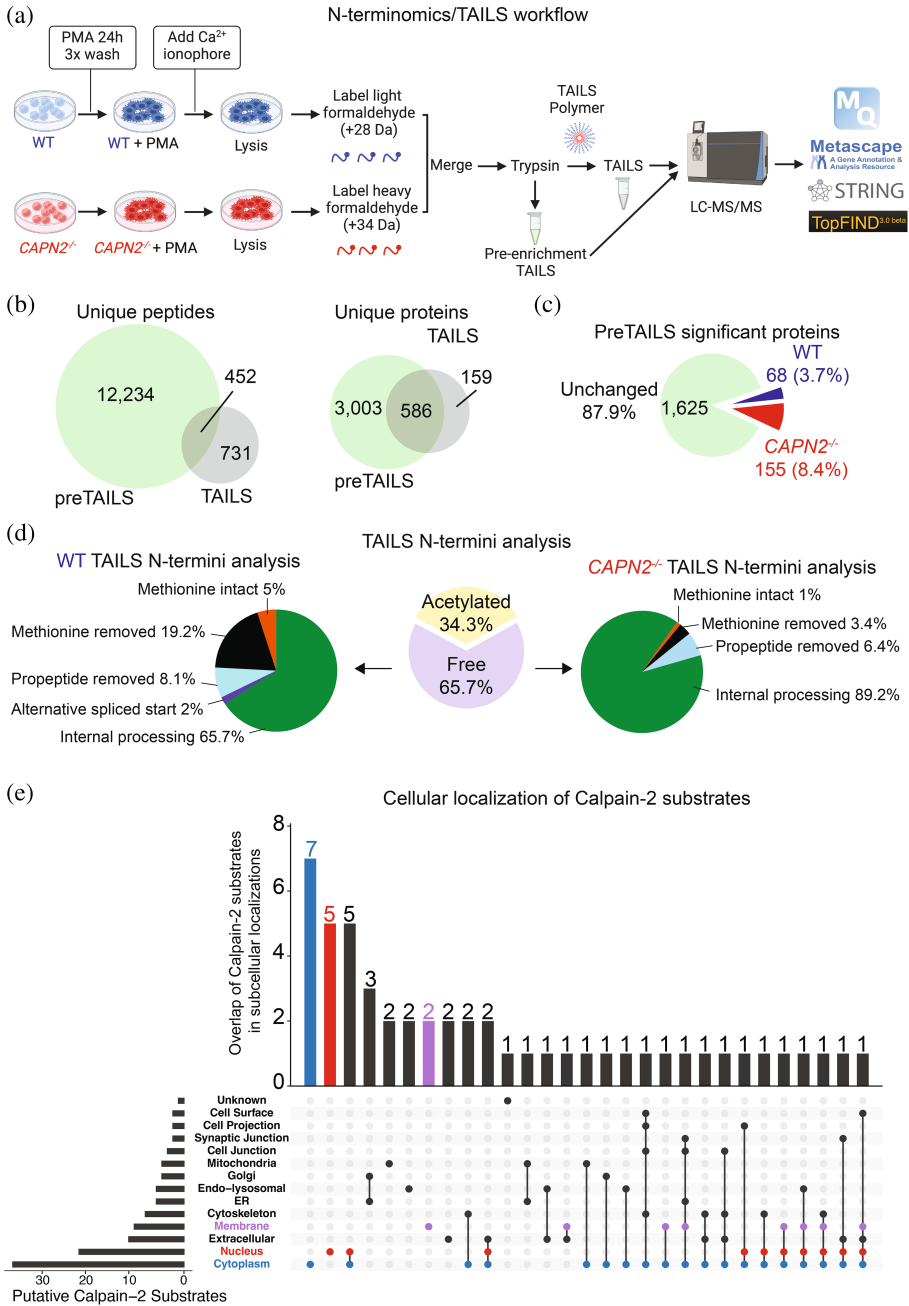
N‐terminomics/TAILS analysis of WT and *CAPN2* ^*−/−*^ THP1 cells. (a) Workflow schematic of N‐terminomics experimental design. PMA‐treated THP‐1 cells (WT or *CAPN2* ^*−/−*^ cells; *n* = 3) were treated with a calcium ionophore to induce Calpain‐2 activation. Proteins were analyzed by shotgun (pre‐TAILS) and N‐terminomics/TAILS analysis. WT proteins were labeled with light formaldehyde (+28 Da) and *CAPN2* ^*−/−*^ cells were labeled with heavy formaldehyde (+34 Da) prior to trypsin digest. N‐termini were enriched by negative selection with the TAILS polymer. Following LC–MS/MS, data was analyzed using Maxquant (Cox and Mann 2008), Metascape (Zhou et al. 2019), STRING‐db (Szklarczyk et al. 2019), and TopFIND (Fortelny et al. 2015; Lange and Overall 2011). (b) The number of unique proteins and peptides identified and shared between preTAILS (shotgun proteomics pre‐enrichment) and TAILS with a false discovery rate (FDR) <1%. For a complete list, see Tables S3–S6. (c) Statistically changing proteins between WT and *CAPN2* ^*−/−*^ cells. For a complete list, see Table S4 and Figure S4c. (d) Left: distribution of N‐terminal peptides in WT cells in the TAILS enrichment. Middle: acetylated and free N‐terminal peptides. Right: distribution of N‐terminal peptides in *CAPN2* ^*−/−*^ cells in the TAILS enrichment. (e) Upset plot of subcellular localizations of the 51 putative Calpain‐2 substrates identified, as cataloged in Uniprot.org. Compartments of interest are highlighted such that red indicates nucleus, blue indicates cytoplasm, and purple indicates membrane.

To identify cleavage events mediated directly by Calpain‐2 or the proteases that it regulates, we treated PMA‐activated THP‐1 cells with a calcium ionophore to activate Calpain‐2, followed by N‐terminomics/TAILS (terminal amine isotopic labeling of substrates) analysis (Figure 2a–d). Cells were lysed and proteins from WT THP‐1 cells were labeled with light formaldehyde (+28 Da dimethylation), while proteins from *CAPN2* ^*−/−*^ THP‐1 cells were labeled with heavy formaldehyde (+34 Da dimethylation) (Figure 2a). Labeled N‐termini were enriched by negative selection with the dendritic polyglycerol aldehyde TAILS polymer (Kleifeld et al. 2010) (Figure 2a). After sample acquisition by LC–MS/MS, data were analyzed using MaxQuant (Cox and Mann 2008) at 1% FDR, and the TAILS analysis yielded 1183 unique peptides (Figures 2b and S4a,b and Tables S3 and S4). Among all N‐termini detected, 34.4% were acetylated while 65.7% were free N‐termini, corresponding to the typical ratios identified in most cell types (Figure 2d, center) (Lange et al. 2014). In WT cells, we identified predominantly internal N‐termini (65.7%), in addition to other proteoforms, including methionine intact (5%), methionine removed (19.2%), propeptide removed (8.1%) and alternative start sites (2%), whereas in *CAPN2* ^*−/−*^ cells, we identified internal N‐termini (89.2%), methionine intact (1%), methionine removed (3.4%), and propeptide removed (6.4%) (Figure 2d and Tables S5 and S6). Fifty‐one cleavage sites were enriched in WT cells compared to the *CAPN2* ^*−/−*^ cells, which indicates proteases cleaved either directly by Calpain‐2 or indirectly by the proteases that it regulates. Among these were known Calpain‐2 substrates, including three based on peptide profiling: Integrin beta‐2 (Pfaff et al. 1999) (^755^K↓S^756^), Talin‐1 (Shinkai‐Ouchi et al. 2016) (^1352^M↓C^1353^; ^2526^R↓Q^2527^), and Vimentin (Shinkai‐Ouchi et al. 2016) (^100^R↓T^101^; ^159^R↓Q^160^; ^321^R↓Q^322^) (Tables 1 and S5). Using information cataloged in UniProt, we found that 31 of the putative substrates are known to localize to the cytoplasm, 19 are found in the nucleus, and 9 are associated with the cell membrane (Figure 2e).

**TABLE 1:** List of Calpain‐2 dependent cleavage events identified in THP‐1 cells.

Uniprot ID	Gene name	Protein name	Calpain‐2 cleavage site(s)
P27348	YWHAQ	14–3‐3 protein theta	27 K A28
Q9H2W6	MRPL46	39S ribosomal protein L46, mitochondrial	32 L A33
P10809	HSPD1	60 kDa heat shock protein, mitochondrial	104 N T105
P26373	RPL13	60S ribosomal protein L13	103R N104
Q969Q0	RPL36AL	60S ribosomal protein L36a‐like	87R C88
P63261	ACTG1	Actin, cytoplasmic 2	18 K A19, 40H Q41, 230A A231, 231A S232, 232S S233, 235S L236, 244D G245
Q9H4A4	RNPEP	Aminopeptidase B	380R Q381
O43776	NARS1	Asparagine‐tRNA ligase, cytoplasmic	425R L426
P27701	CD82	CD82 antigen	174C S175
P35606	COPB2	Coatomer subunit beta′	189 N C190
P08174	CD55	Complement decay‐accelerating factor	34G D35
P28838	LAP3	Cytosol aminopeptidase	440R Q441
Q96EP5	DAZAP1	DAZ‐associated protein 1	373Y G374
P33993	MCM7	DNA replication licensing factor MCM7	202P L203
Q01469	FABP5	Fatty acid‐binding protein, epidermal	67C T68
P09972	ALDOC	Fructose‐bisphosphate aldolase C	116 L A117, 118G T119
P06396	GSN	Gelsolin	271 L Q272, 510 T A511
P28799	GRN	Granulins	280G D281, 517 K D518
P11142	HSPA8	Heat shock cognate 71 kDa protein	221S T222
P04792	HSPB1	Heat shock protein beta‐1	79R Q80
P08238	HSP90AB1	Heat shock protein HSP 90‐beta	458H T459
P14866	HNRNPL	Heterogeneous nuclear ribonucleoprotein L	228R K229
P22626	HNRNPA2B1	Heterogeneous nuclear ribonucleoproteins A2/B1	247Y G248, 325R N326
Q99878	HIST1H2AJ	Histone H2A type 1‐J	59 L T60
P12268	IMPDH2	Inosine‐5′‐monophosphate dehydrogenase 2	245 L C246
P05107	ITGB2	Integrin beta‐2	755 K S756
P08700	IL3	Interleukin‐3	57 N N58
P33121	ACSL1	Long‐chain‐fatty‐acid‐CoA ligase 1	240R C698
O00754	MAN2B1	Lysosomal alpha‐mannosidase	601 W S602, 985 L D986
P40121	CAPG	Macrophage‐capping protein	6P Q7
Q14697	GANAB	Neutral alpha‐glucosidase AB	187S Q188
Q15233	NONO	Non‐POU domain‐containing octamer‐binding protein	409G T410
P67809	YBX1	Nuclease‐sensitive element‐binding protein 1	162Y Q163
O95153	TSPOAP1	Peripheral‐type benzodiazepine receptor‐associated protein 1	376Q N377
Q15365	PCBP1	Poly(rC)‐binding protein 1	297R Q298
Q15652	JMJD1C	Probable JmjC domain‐containing histone demethylation protein 2C	288A M289
Q8WUM4	PDCD6IP	Programmed cell death 6‐interacting protein	727Y Q728
P49257	LMAN1	Protein ERGIC‐53	307F Q308
Q8NFI4	ST13P5	Putative protein FAM10A5	15 M C16
Q9NQC3	RTN4	Reticulon‐4	157P V158
P13489	RNH1	Ribonuclease inhibitor	205 K L206
Q01844	EWSR1	RNA‐binding protein EWS	254Y S255
Q96BR1	SGK3	Serine/threonine‐protein kinase Sgk3	6 T M7
Q9Y490	TLN1	Talin‐1	1352 M C1353, 2526R Q2527
Q96FV9	THOC1	THO complex subunit 1	600R Q601
Q13263	TRIM28	Transcription intermediary factor 1‐beta	579 M A580, 580A L581
O14773	TPP1	Tripeptidyl‐peptidase 1	195G L196
Q71U36	TUBA1A	Tubulin alpha‐1A chain	202F M203
P43405	SYK	Tyrosine‐protein kinase SYK	313R Q314
O75396	SEC22B	Vesicle‐trafficking protein SEC22b	133R N134
P08670	VIM	Vimentin	100R T101, 159R Q160, 321R Q322

**FIGURE 3:**
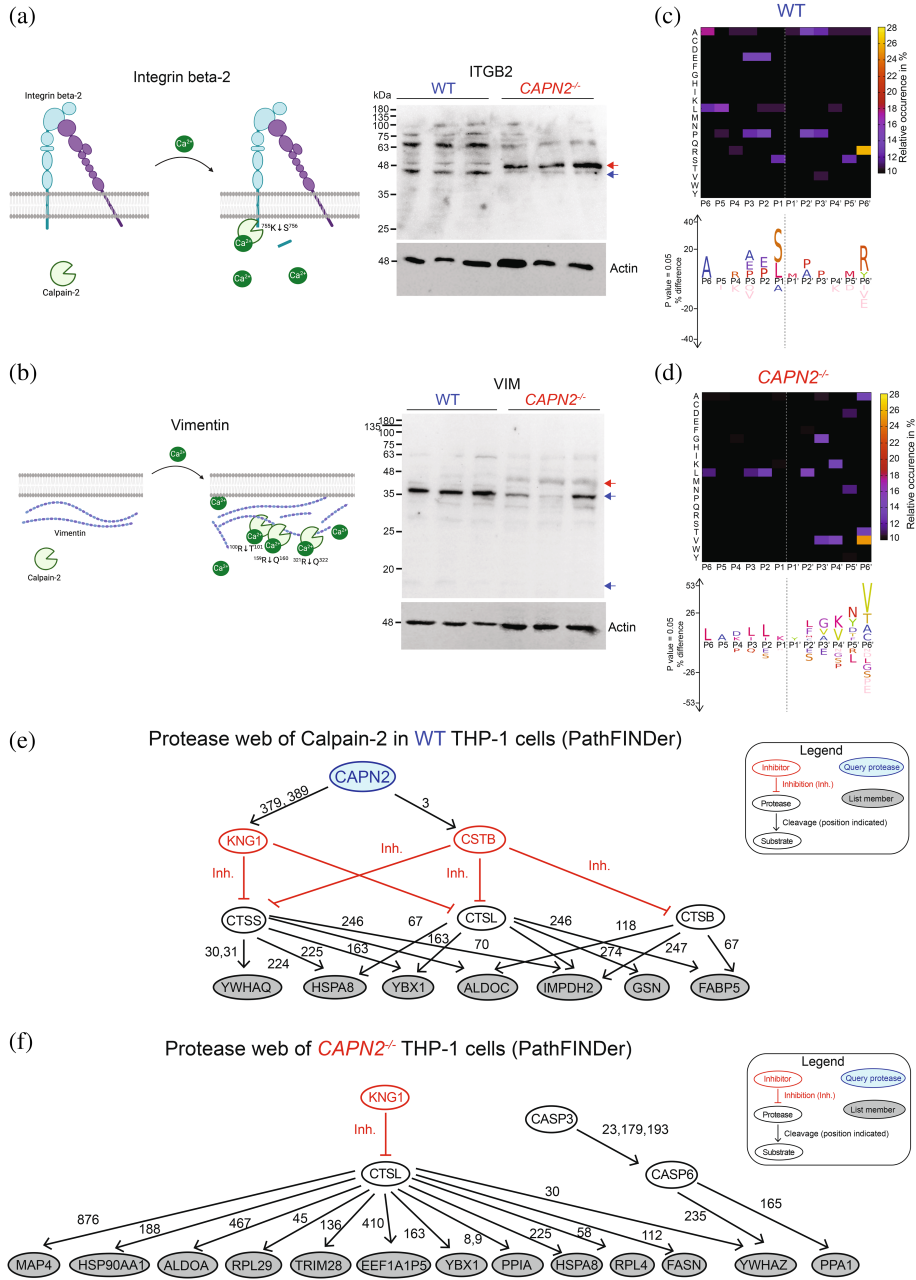
Cleavage specificity and PathFINDer Analysis of WT and *CAPN2* ^*−/−*^ THP1 cells. (a) Left, schematic representation of the cleavage of integrin beta‐2 in the presence of Calpain‐2. Right, Western blot validation of integrin beta‐2 in WT and *CAPN2* ^*−/−*^ THP‐1 cells. The blue arrow shows where the cleavage occurs in WT cells (*n* = 3). The red arrow shows the uncleaved form of integrin beta‐2 detected in *CAPN2* ^*−/−*^ THP‐1 cells (*n* = 3). (d) Left, schematic representation of the cleavage of Calpain‐2‐dependent cleavage of vimentin. Right, Western blot validation of vimentin in WT and *CAPN2* ^*−/−*^ THP‐1 cells. The blue arrow shows where the cleavage occurs in WT cells (*n* = 3). The red arrow shows the uncleaved form of vimentin detected in *CAPN2* ^*−/−*^ THP‐1 cells (*n* = 3). (c) Upper panel, cleavage sites identified in WT THP‐1 cells are depicted as heat maps from P6 to P6′ residues using WebPICS (Schilling et al. 2011). Yellow: upregulated. Black: no enrichment/preference. Lower panel, peptide sequence profiles of significantly elevated neo‐N‐terminal peptides in WT THP‐1 cells identified in the TAILS analysis using IceLogo (Colaert et al. 2009). Significantly (*p* < 0.05) overrepresented amino acids are shown above, and underrepresented residues are shown below the x‐axis. Statistical analysis was determined by a two‐tailed unpaired Student's *t* test and was adjusted for multiple comparisons. (d) Upper panel, cleavage sites identified in *CAPN2* ^*−/−*^ THP‐1 cells are depicted as heat maps from P6 to P6′ residues using WebPICS (Schilling et al. 2011). Yellow: upregulated. Black: no enrichment/preference. Lower panel, peptide sequence profiles of significantly elevated neo‐N‐terminal peptides in *CAPN2* ^*−/−*^ THP‐1 cells identified in the TAILS analysis using IceLogo (Colaert et al. 2009). Significantly (*p* < 0.05) overrepresented amino acids are shown above, and underrepresented residues are shown below the x‐axis. Statistical analysis was determined by a two‐tailed unpaired Student's *t* test and was adjusted for multiple comparisons. (e) Protease web analysis using PathFINDer (Fortelny et al. 2015) for WT THP‐1 cells. Calpain‐2 (CAPN2) is the query protease shown in blue. Inhibitors are shown in red. Substrates are shown in black. Proteins identified by the TAILS analysis are shown in gray. (f) Protease web analysis using PathFINDer (Fortelny et al. 2015) for *CAPN2* ^*−/−*^ THP‐1 cells. Inhibitors are shown in red. Substrates are shown in black. Proteins identified by the TAILS analysis are shown in gray.

To validate selected putative cleavage sites, we used an immunoblotting approach. Cleavage of integrin beta‐2 at ^755^K↓S^756^ would result in the removal of 10 amino acids from the C terminus of the protein (Figure 3a, left panel). By blot, we observed a shift in the molecular weight of ITGB2 by ~2000 Da in the absence of CAPN2, which supports cleavage at this site (Figures 3a, left and S2a,b). Densitometry analysis revealed a statistically significant difference between full length and cleaved ITGB2 between WT and *CAPN2* ^*−/−*^ cells (Figure S2c). We also detected statistically significant differences in vimentin processing between WT and *CAPN2* ^*−/−*^ cells, reflecting differential cleavage at multiple sites (Figures 3b and S2d–f).

Next, we examined differences in cleavage site preferences between WT and *CAPN2* ^*−/−*^ cells by generating IceLogos of the cleavage sites enriched in each genotype (P6‐P6′) (Colaert et al. 2009). In the WT cells, we identified an enrichment of serine and leucine residues at P1, alanine in P6, methionine in P1′ and arginine at P6′ (Figure 3c). In *CAPN2* ^*−/−*^ cells, we observed enriched lysine at P1, tyrosine at P1′, and valine at P6′ (Figure 3d). Next, we used TopFINDER (Fortelny et al. 2015) to identify other potential proteases that can cleave the putative substrates we identified. In WT cells, we found that other proteases such as Cathepsin S could be responsible for six cleavages and Cathepsin B and L could be responsible for four cleavages (Pang et al. 2024) (Figure S3e,f). In *CAPN2* ^*−/−*^ cells, we found that Cathepsin L could be responsible for 14 cleavages, Cathepsin S for 12 cleavages and Granzyme B for 10 cleavages (Figure S3a,b). Next, we use PathFINDER (Fortelny et al. 2015) to integrate the N‐termini of all proteins in a protease web where Calpain‐2 (CAPN2) was the query protease in WT cells (Figure 3e) and no query protease in *CAPN2* ^*−/−*^ cells (Figure 3f). All known cleavage sites (number beside each arrow) were integrated between Calpain‐2 (CAPN2; blue), the protease inhibitors (red), identified hits from our N‐terminomics/TAILS data (gray) and known proteolytic processing from the literature (white) (Figure 3e). In WT cells, we identified a potential role of Cathepsin B, L and S, whereas in *CAPN2* ^*−/−*^ cells, we identified a potential role of Cathepsin L, Caspase‐3 and ‐6 (Figure 3e,f).

### 2.3 The expression of Calpain‐2 and its substrates in clinical samples of patients with acute myeloid leukemia

**FIGURE 4:**
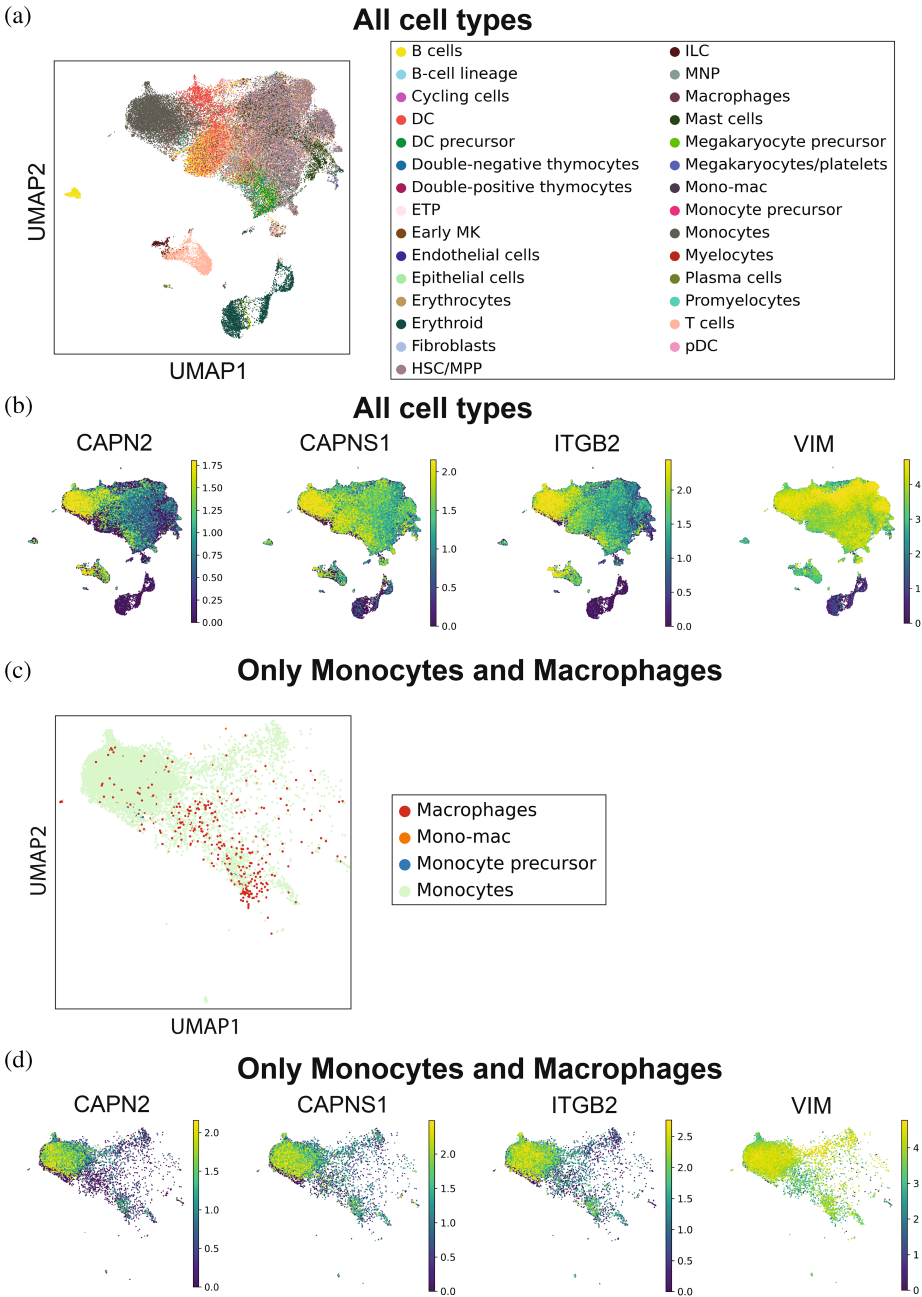
Single‐cell RNA‐sequencing and Western blot analyses in acute myeloid leukemia (AML) patients and THP‐1 cells. (a) Cell annotation using the cell‐type method. UMAP shows 32 cell clusters in the bone marrow (*n* = 6) and peripheral blood (*n* = 6) of AML patient biopsies (GSE241989). (b) The expression pattern of *CAPN2*, *CAPNS1*, *ITGB2*, *SYK*, and *VIM* in all clusters using UMAP. (c) Cell annotation using the cell‐type method showing only the monocytes and macrophages cell clusters in the bone marrow (*n* = 6) and peripheral blood (*n* = 6) of AML patient biopsies (GSE241989). (d) The expression pattern of *CAPN2*, *CAPNS1*, *ITGB2*, *SYK*, and *VIM* in all clusters using UMAP of the monocytes and macrophages cell clusters.

Although THP1 cells are broadly used as a model cell line to study macrophage functions, these cells were originally derived from a patient with acute monocytic leukemia (AML). Therefore, we wanted to also explore if patients with acute monocytic leukemia also express Calpain‐2 and its substrates in the context of biopsies taken from AML patients and healthy controls. We mined the literature for human diseases modeled by THP‐1 cells and identified a single‐cell RNA sequencing (scRNA‐seq) dataset (GSE241989) obtained from primary human AML, including six bone marrow (BM) and six peripheral blood (PB) samples (Bordeleau et al. 2024). We analyzed the population distribution of scRNA‐seq data of bone marrow‐derived cells and peripheral blood isolated from AML patients, and uniform manifold approximation and projection (UMAP) based clustering identified 79 individual cell clusters (Figure 4a,b). Focusing our analysis on only monocytes, macrophages, and monocyte precursors, we observed that *CAPN2* was expressed predominantly by monocytic cell populations in addition to macrophages (Figure 4c,d). *CAPNS1*, as well as the two substrates *ITGB2* and *VIM*, were also mainly expressed by monocytes and macrophages (Figure 4c,d), providing evidence that these proteins could be cleaved in a Calpain‐2‐dependent manner in the landscape of human AML.

### 2.4 Comparison of the proteomes of wild type and Calpain‐2‐deficient THP‐1 cells

**FIGURE 5:**
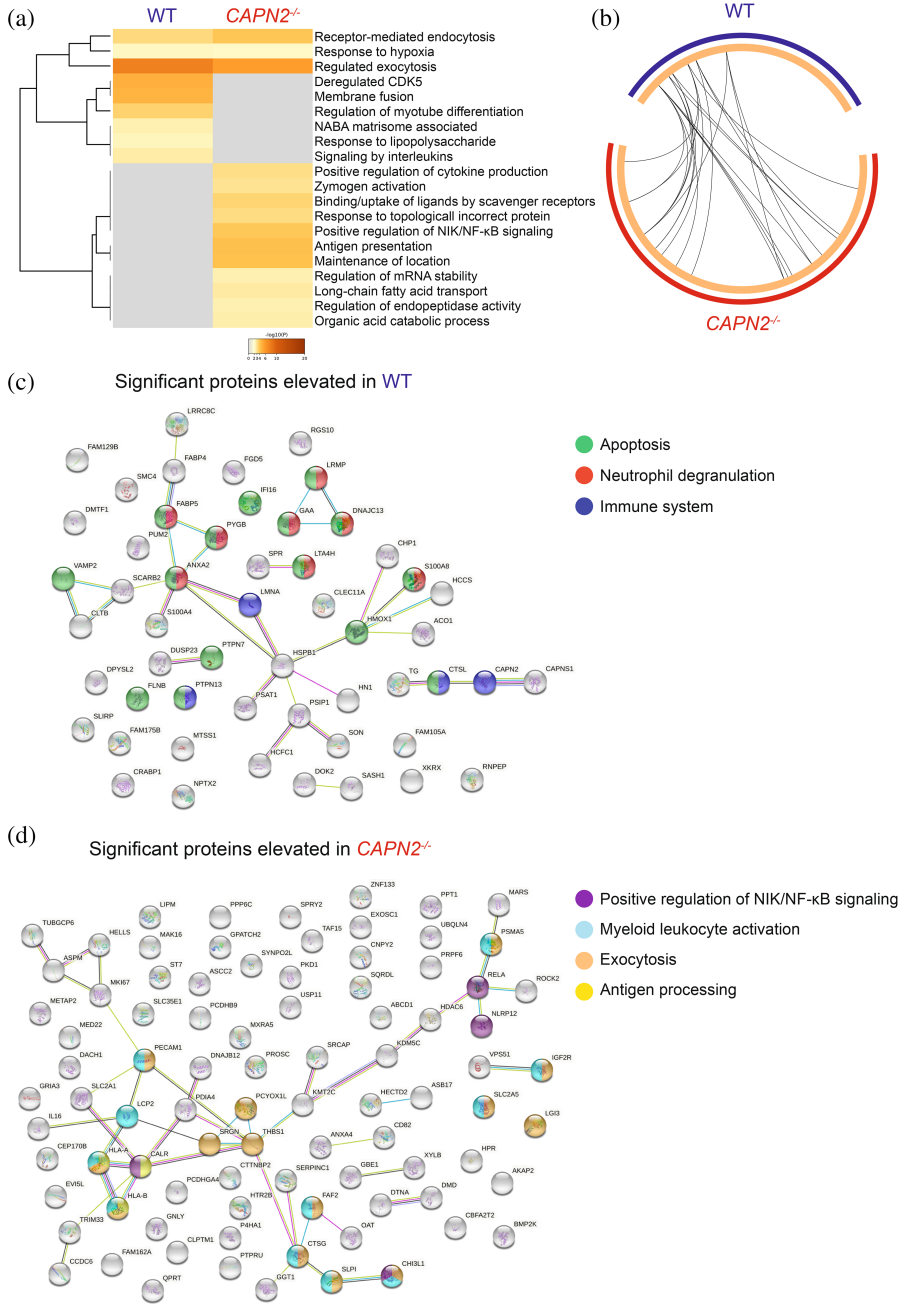
Proteomics analysis of human WT and *CAPN2* ^*−/−*^ THP‐1 cells treated with PMA and activated with a calcium ionophore. (a) Pre‐TAILS enrichment data from the workflow in Figure 2a was analyzed by Metascape (Zhou et al. 2019) to reveal differential pathways between WT and *CAPN2* ^*−/−*^ THP‐1 cells treated with PMA and a calcium ionophore. Accumulative hypergeometric *p*‐values and enrichment factors were calculated and used for filtering. Remaining significant terms were then hierarchically clustered into a tree based on Kappa‐statistical similarities among their gene's memberships. Then, 0.3 kappa score was applied as the threshold to cast the tree into term clusters. (b) Circos plot indicating the overlapping Gene Ontology (GO) terms between WT and *CAPN2* ^*−/−*^ THP‐1 cells treated with PMA and a calcium ionophore. Data was analyzed from three biological replicates. (c) STRING‐db (Szklarczyk et al. 2019) analysis of WT THP‐1 cells treated with PMA and a calcium ionophore. Enrichment of apoptosis (green), neutrophil degranulation (red), and immune system (blue) are shown in colored spheres. (f) STRING‐db (Szklarczyk et al. 2019) analysis of *CAPN2* ^*−/−*^ THP‐1 cells treated with PMA and a calcium ionophore. Enrichment of positive regulation of NIK/NF‐κB signaling (purple), myeloid leukocyte activation (pale blue), exocytosis (orange), and antigen processing (yellow) are shown in colored spheres.

In addition to substrate analysis in the presence of active Calpain‐2 (after treatment with the Ca^2+^ ionophore; TAILS), we also analyzed the pre‐enrichment fraction to identify protein level changes dependent on active Calpain‐2 (Figure 2a; pre‐TAILS). We identified 12,485 unique peptides corresponding to 3589 unique proteins, including 68 that were enriched in WT cells and 155 that were enriched in *CAPN2* ^*−/−*^ cells (Figures 2b,c and S4a–c and Table S4). Using Metascape (Zhou et al. 2019) analysis, we observed an enrichment in NABA matrisome, response to lipopolysaccharide, and signaling by interleukins among proteins increased in WT cells. In the *CAPN2* ^*−/−*^ cells, we identified an enrichment of proteins associated with NIK/NF‐κB signaling, antigen presentation, long‐chain fatty acid transport, and organic acid catabolic process (Figure 5a). Although we identified key differences in pathway enrichment and Gene Ontology (GO) terms, there was some significant overlap, as shown by a Circos plot (Figure 5b), between WT and *CAPN2* ^*−/−*^ cells including receptor‐mediated endocytosis, response to hypoxia, and regulated exocytosis. Using STRING‐db (Szklarczyk et al. 2019), there was an enrichment of apoptosis, neutrophil degranulation, and immune system in the WT cells, along with an enrichment of Cathepsin L (CTSL) that further validates our PathFINDer analysis in Figure 3e. As expected, Calpain‐2 was reduced in the *CAPN2* ^*−/−*^ cells, but in addition, we observed elevation of Calpain small subunit 1 (CAPNS1) which is required for Calpain‐2 activation (Figure 5c). Using STRING‐db (Szklarczyk et al. 2019) on the significant proteins elevated in *CAPN2* ^*−/−*^ cells, there was an enrichment of NIK/NF‐κB signaling, myeloid leukocyte activation, exocytosis, and antigen presentation (Figure 5d).

### 2.5 Calpain‐2 regulates pyrimidine and glutathione metabolism

**FIGURE 6:**
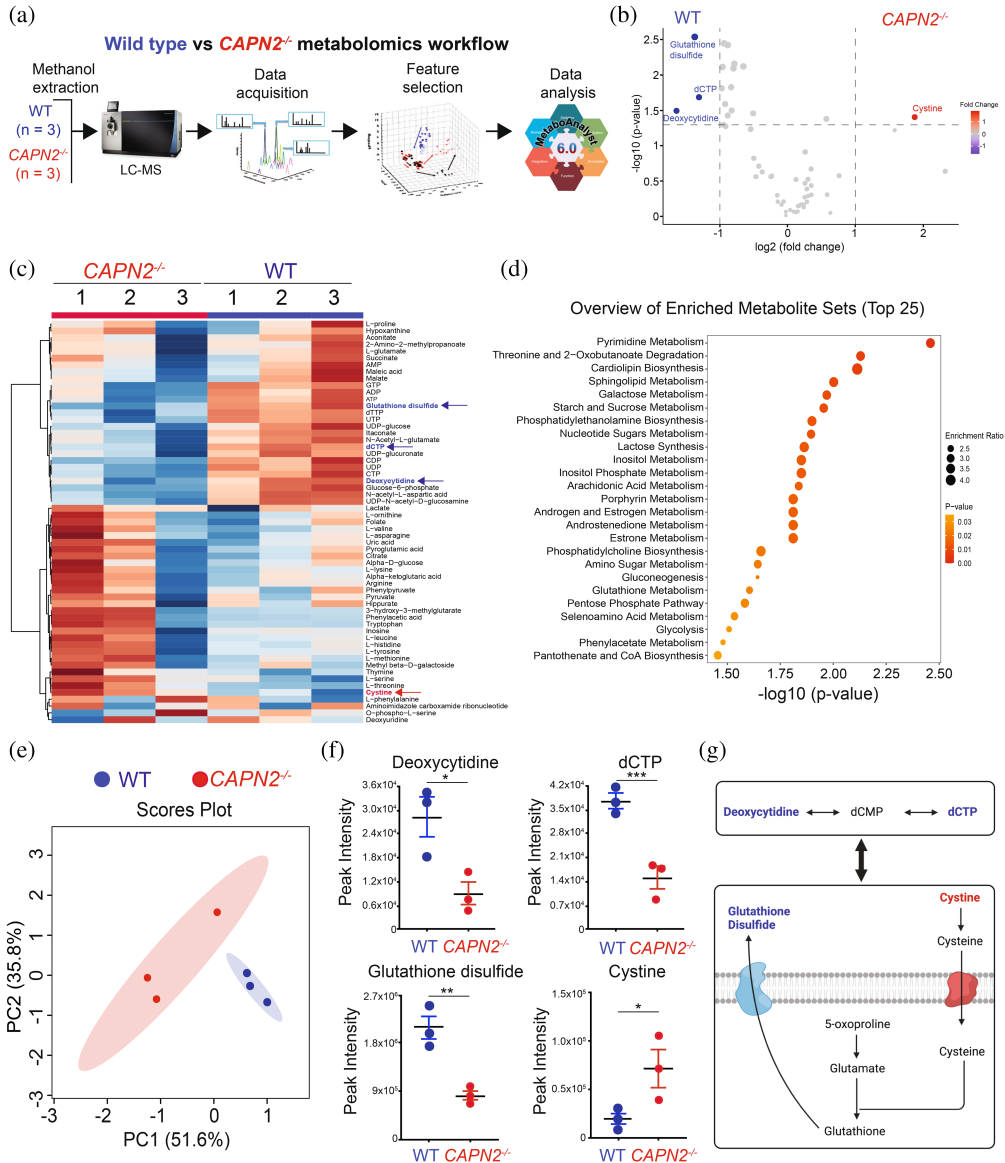
Metabolomics analysis of human WT and *CAPN2* ^*−/−*^ THP‐1 cells treated with PMA and activated with a calcium ionophore. (a) Workflow schematic of metabolomics experimental design in WT and *CAPN2* ^*−/−*^ cells (*n* = 3). (b) Volcano plot of the metabolomics data. Metabolites shown in blue are significantly different in WT cells while those changing in *CAPN2* ^*−/−*^ cells are shown in red. Significance was determined by an interquartile boxplot analysis and an adjusted Student's *t* test. (c) Heatmap of the metabolomics data. Metabolites shown with a blue arrow are significantly changing in WT cells and those depicted with a red arrow are changing in *CAPN2* ^*−/−*^ cells. (d) Enrichment analysis of upregulated metabolites computed by Hits/Expected (hits = observed hits; expected = expected hits) of WT and *CAPN2* ^*−/−*^ cells. Data were analyzed using MetaboAnalyst 6.0, Small Molecule Pathway Database (SMPDB) metabolite set library. (e) Principal component analysis (PCA) was performed on the metabolomics data. (f) Deoxycytidine, dCTP, glutathione disulfide and cystine relative abundance in WT and *CAPN2* ^*−/−*^ cells determined by LC–MS. Data are represented as bar graph and analyzed by two‐tailed paired t test: *p < 0.05, **p < 0.01, ***p < 0.001 (*n* = 3). (g) Schematic representation of the metabolomic pathway enrichment in WT and *CAPN2* ^*−/−*^ cells. Metabolites significantly elevated in WT cells are shown in blue and in *CAPN2* ^*−/−*^ cells are shown in red.

In our quantitative proteomics analysis (Figure 5), we identified enrichment in metabolic processes (long‐chain fatty acid transport and organic acid catabolism) in *CAPN2* ^*−/−*^ cells. We therefore performed metabolomics to identify Calpain‐2‐dependent changes in metabolites (Figure 6a). We profiled 59 metabolites and identified a significant elevation of dCTP, deoxycytidine, and glutathione disulfide in WT cells and a significant elevation of cystine in *CAPN2* ^*−/−*^ cells (Figure 6b,c). Analysis with Metaboanalyst (Pang et al. 2024) revealed pyrimidine metabolism, threonine and 2‐oxobutanoate degradation, and cardiolipin biosynthesis as the most enriched and significant pathways (Figure 6d). Specifically, in WT cells, dCTP, deoxycytidine, and glutathione disulfide are linked with an enrichment in pyrimidine and glutathione metabolism (Figure 6d). Using a Principal Component Analysis (PCA), we identified the two most valuable variables that helped to simplify the complexity of the dataset, separating features between cell lines (Figure 6e). We also performed statistical analysis of all samples to determine significance and reproducibility; this resulted in the identification of four significantly changed metabolites: dCTP, deoxycytidine, and glutathione disulfide in WT cells and cystine in *CAPN2* ^*−/−*^ cells (Figure 6f). This suggests that Calpain‐2 is critical to sustain the metabolic regulation of THP‐1 cells via deoxycytidine, dCTP, and glutathione disulfide, and when absent, cystine metabolite was elevated (Figure 6g). As shown previously, AML cells are sensitive to various metabolism inhibitors including depletion of L‐cyst(e)ine (Cramer et al. 2017), inhibitors of pyrimidine nucleotide biosynthesis (Mishra et al. 2023), and several others (Krauss et al. 2019). Interestingly, cystine levels were elevated in *CAPN2* ^*−/−*^ cells, and cystine dependency was demonstrated to be elevated in patients with AML and a poor prognostic marker (Pardieu et al. 2022). Therefore, Calpain‐2 might be implicated as either a direct or indirect effector in THP‐1 cell metabolic regulation.

## 3 DISCUSSION

Calpain‐1 and ‐2 have been implicated as causal proteases in various diseases including cataract formation, myocardial infarction, multiple sclerosis, stroke, cancer, and Parkinson's disease and thus represent potential therapeutic targets (Zatz and Starling 2005). In this manuscript, we aimed to analyze, for the first time ever using an unbiased N‐terminomics approach, Calpain‐2‐dependent proteolysis. THP‐1 cells are broadly used as a culture model of macrophages; however, these cells were originally derived from a patient with AML. As cancer cells have a higher metabolic need than most cells, we also aimed to characterize their metabolome and their proteome to identify potential links with Calpain‐2 substrates and metabolism or an increase in metabolic proteins. To assure that these cells also expressed our newly identified putative Calpain‐2 substrates, we additionally mined the literature and profiled another dataset taken from AML patients and healthy controls to connect our experiments to primary patient samples. To characterize the biological function of a protease, it is critical to identify the repertoire of substrates it can cleave. Here, we sought to identify the unique Calpain‐2 substrates in PMA‐activated human THP‐1 cells stimulated with a calcium ionophore. We used a genetic approach to knock out Calpain‐2. Next, we identified that upon PMA activation of THP‐1 cells, there was varying abundance of other proteases and protease inhibitors between WT and *CAPN2* ^*−/−*^ cells. This is evidence that Calpain‐2 indirectly or directly impacts changes of the protease web (Esser‐Skala and Fortelny 2022; Fortelny et al. 2014; Keller et al. 2007), described as a system biology network where proteases, their substrates, and protease inhibitors act in concerted networks. For example, the Calpain‐2 protease web is connected with Aminopeptidase N, Matrix metalloproteinase‐9, Cathepsin G, and Cathepsin D when activated with PMA (Figure 1b,c). Upon treatment with PMA and a calcium ionophore, Calpain‐2 was active (with the presence of a calcium ionophore), and the protease web was modified and regulated via Cystatin B, Cathepsin B, L, and S (Figure 2c). In the *CAPN2* ^*−/−*^ cells, the protease web included Caspase‐3, Caspase‐6, and Cathepsin L (Figure 2d).

Using an unbiased proteomics and N‐terminomics/TAILS approach to characterize protease substrates, we identified 51 potential Calpain‐2 substrates, including 2 known substrates based on peptide profiling: Integrin beta‐2 (Pfaff et al. 1999) (^755^K↓S^756^) and Talin‐1 (Shinkai‐Ouchi et al. 2016) (^1352^M↓C^1353^; ^2526^R↓Q^2527^). However, these substrates have not been identified in THP‐1 cells, and their exact cleavage sites were unknown; therefore, N‐terminomics/TAILS is a valid approach to identify new Calpain substrates. Importantly, it is possible that not all 51 putative substrates are direct substrates of Calpain‐2, as they might be cleaved by other proteases that could be activated by Calpain‐2. Therefore, every putative substrate would need to be carefully validated one by one, which goes beyond the scope of this current work.

When we compared the proteome of WT and *CAPN2* ^*−/−*^ cells, we identified an enrichment of signaling by interleukins in WT cells (Figure 5a), which potentially correlates with IL‐3 being a putative substrate of Calpain‐2 (Table 1). We also found an enrichment of proteins associated with the immune system, apoptosis, and neutrophil degranulation (Figure 5c). In *CAPN2* ^*−/−*^ cells, we identified an enrichment of long‐chain fatty acid transport (metabolism), antigen processing, and myeloid leukocyte activation (Figure 5a,d). This led us to investigate if the metabolites between WT and *CAPN2* ^*−/−*^ cells were different (Figure 6a). We identified an enrichment of deoxycytidine, dCTP, and glutathione disulfide in WT cells and cystine in *CAPN2* ^*−/−*^ cells, suggesting changes in the pyrimidine, threonine, and 2‐ocobutanoate degradation, and cardiolipin biosynthesis metabolic pathways (Figure 6b,d,f). Our data align with other studies that demonstrated a key role of cardiolipin in the inflammatory metabolic reprogramming of macrophages (Reynolds et al. 2023), and the role of pyrimidine in leukocytes and leukemic cells (Siddiqui and Ceppi 2020).

Importantly, although THP1 cells are broadly used model cell line to study macrophage functions, it was originally a cell line derived from a patient with acute monocytic leukemia. Therefore, our results could be applicable to macrophage biology but also AML pathology. We validated selected Calpain‐2 substrates and verified their co‐expression with Calpain‐2 in monocytes obtained from human AML samples, including ITGB2 and VIM (Figure 4a,b). We validated that, in THP‐1 cells, ITGB2 and VIM are present in the same cells that produce CAPN2 and Calpain small subunit CAPNS1 required for Calpain‐2 activity (Figure 4). We also demonstrated that *CAPN2* ^*−/−*^ cells exhibit differential cleavage of integrin beta‐2 and vimentin as compared to WT cells (Figure 3a,b). Collectively, our data present new evidence of previously unknown Calpain‐2‐mediated cleavage events. It also demonstrates an important metabolic role regulated in part by Calpain‐2. Our study may point towards new mechanistic understanding of Calpain‐2 in monocytes, macrophages, and leukemic cells that might be altered in diseases.

## 4 MATERIALS AND METHODS

### 4.1 Cell culture

THP‐1 cells were obtained from the lab of Dr. Robin Yates at the University of Calgary. Cells were cultured in THP‐1 cell culture media: RPMI 1640, 10 mM L‐glutamine, 10 mM HEPES, and 10% fetal bovine serum (Thermo Fisher Scientific, Waltham, MA). Cells were then incubated at 37°C and 5% CO_2_ in cell culture flasks and maintained at 5–8 × 10^5^ cells/mL. All experiments were performed on cells between passage numbers 3–8.

### 4.2 Activation with phorbol myristate acetate and calcium ionophore

To achieve a “macrophage‐like” phenotype, THP‐1 cells were differentiated and activated with Phorbol 12‐myristate 13‐acetate (PMA) (Sigma, P‐8139) (Lund et al. 2016). Cells were seeded at a density of 1 × 10^6^ cells/mL in a T175 culture flask and PMA (50 nM) was added for 18 h at 37°C in 5% CO_2_. After the cells became adherent, PMA was removed and replaced with fresh media. Calcium ionophore (50 nM; A23187) (Sigma, C7522) was added and cells were incubated for 1 h. The media was then discarded, and cells were washed three times with PBS and lysed as described below.

### 4.3 Generation of CRISPR *CAPN2* ^−/−^ THP‐1 cells

gRNA for Calpain‐2 was generated as described in the CRISPR design guide by the Zhang lab (crispr.mit.edu) and cloned into the lentiCRISPRv2 plasmid (Addgene plasmid # 98290; http://n2t.net/addgene:98290; RRID:Addgene_98,290). Cloning was performed as described by Shalem et al. (2014), and lentivirus was generated after transfecting the *CAPN2* targeted‐gRNA lentiCRISPRv2 construct with the psPAX2 and pCMV‐VSVG plasmids into HEK293T cells (Addgene plasmid # 12260; http://n2t.net/addgene:12260; RRID:Addgene_12,260 and Addgene plasmid # 8454; http://n2t.net/addgene:8454; RRID:Addgene_8454), respectively. Viral products were harvested from the supernatant and centrifuged at 122,000*g* for 2 h. The concentrated virus was applied to THP‐1 cells and transduced cells were selected using puromycin. Surviving cells were plated by limiting dilution method to derive monoclonal cell lines from single clones and each replicate was a different clone. The WT cells also underwent a sham treatment.

### 4.4 Proliferation counting assay

WT and *CAPN2* ^*−/−*^ THP‐1 cells (2000 cells) were seeded into a 12‐well plate. Cell counts were recorded at time 0, 24, and 48 h using a hemocytometer.

### 4.5 Western blotting

Cells were lysed and mechanically scraped using 1 mL lysis buffer (1% SDS, 10 mM EDTA, 7.8 μg/mL ammonium bicarbonate, and protease inhibitor tablets (Roche cOmplete) in high‐performance liquid chromatography water, pH 8.0). Lysates were transferred to 2 mL Eppendorf tubes and sonicated to shear DNA (3× for 5 s) using a probe tip sonicator (Microson ultrasonic cell disruptor). Lysates were then centrifuged at 14,000*g* for 10 min in a microcentrifuge pre‐cooled to 4°C. The supernatant was collected, and the protein concentration was measured using a Nanodrop (Implen, NanoPhotometer (N50)). Samples were then boiled at 100°C for 5 min. SDS‐PAGE gels were run at 80 V until the dye front passed the stacking gel and then at 140 V until the dye front reached the bottom of the gel. The stacking gel was removed, and the resolving gel was prepared for transfer. Polyvinylidene fluoride (PVDF) (Immobilon®) membranes were presoaked in 100% methanol for 5 min and placed between two 1 mm sheets of blot paper that had been folded three times (3 mm high) and soaked in transfer buffer (30 g Tris‐base, 144 g Glycine, 1 L H_2_O to make 10×, then 10% methanol in 1×). The resolving gel was placed between the membrane and one of the 3 mm folded sheets, and the 4‐layer ensemble was mounted on a protein transfer unit. The transfer was performed at 100 V for 1 h on ice. The PVDF membrane with transferred proteins was incubated in a blocking solution (3% w/v Bovine serum albumin; Alfa Aesar, J64655) in TBS‐Tween 0.1% v/v for 1 h at room temperature on a shaker. The primary antibody was dissolved in 1% BSA (w/v) in double‐distilled water and added to the membrane overnight at 4°C. Primary antibodies used include RP1 CAPN2 antibody (Triple Points Biologic), Integrin Beta‐2 (Abbiotec: 251165), and Vimentin (Boster: pb9359). The membrane was washed 5–6 times with TBST for 7 min, and the secondary antibody, Goat anti Mouse (Columbia Bioscience: HRP‐112) conjugated to Horse Radish Peroxidase (HRP) diluted in 1% BSA, was added for 1 h at room temperature on a shaker. The membrane was then washed 5–6 times with TBST for 7 min. A chemiluminescence detection kit was used according to the manufacturer's instructions to develop the blot, and blots were visualized using the ChemiDoc MP Imaging System (BioRad).

### 4.6 Proteomics sample preparation

Unstimulated and PMA‐stimulated THP‐1 cells were lysed using 1 mL lysis buffer (1% SDS, 10 mM EDTA, 7.8 mg/mL ammonium bicarbonate and protease inhibitor tablets (Roche cOmplete) in high‐performance liquid chromatography water (HPLC water), pH 8.0) and mechanically scraped. Cell contents were collected in a 2 mL Eppendorf tube and sonicated to shear DNA. Lysates were then centrifuged at 14,000*g* for 10 min in a microcentrifuge pre‐cooled to 4°C to pellet DNA. The supernatant was collected, and the protein concentration was measured using a Nanodrop (Implen, NanoPhotometer (N50)).

### 4.7 Shotgun quantitative proteomics

For shotgun proteomics analysis, 400 μg of protein was diluted in 500 mL lysis buffer in a LoBindTM Eppendorf tube. Ten microliter of dithiothreitol (DTT) (0.154 g/mL, prepared fresh in HPLC water) was added to the solution and incubated at 37°C for 1 h, then 10 μL iodoacetamide (IAA) (0.0925 g/mL) was added at room temperature for 30 min while protecting from light. The reaction was quenched by further adding 10 μL of DTT for 30 min at room temperature. To precipitate the protein, 250 μL of 50% trichloroacetic acid (TCA) was added to each sample on ice; then all were incubated at 4°C for 30 min. Tubes were then centrifuged at 14,000*g* for 10 min, then the supernatant was removed and replaced with ice‐cold acetone, leaving the pellet undisturbed. The samples were centrifuged again at 14,000*g* for 5 min, acetone was discarded, and then the acetone wash was repeated another 3 times. After the last acetone wash, the pellet was resuspended in 50 μL of 100 mM NaOH and trypsinized using 10 μg of trypsin at pH 8.0 at 37°C overnight. The next day, WT samples were labeled by adding 22 mL of 20 mM 37% heavy formaldehyde (CD_2_O: 13C, D2 solution; +34 Da), followed by 22 mL of 1M sodium cyanoborohydride (NaBH_3_CN) prepared fresh in fume hood using HPLC water to a final concentration of 10 mM. *CAPN2* ^*−/−*^ samples were labeled in the same way but using 20% light formaldehyde (CH_2_O; +28 Da). All samples were pH‐balanced to 6.5 using 1M HCl and incubated at 37°C for 2–3 h. Heavy‐ and light‐labeled samples were combined into one tube, and SEP PAK C18 chromatography was performed as recommended by the vendor.

### 4.8 N‐terminomics/TAILS and shotgun proteomics of THP‐1 cells

WT and *CAPN2* ^*−/−*^ THP‐1 cells (*n* = 3) were treated with PMA overnight followed by a 1 h treatment with the calcium ionophore (A23187; Sigma, C7522). Samples were lysed (1% SDS, 10 mM EDTA, 7.8 mg/mL ammonium bicarbonate and protease inhibitor tablets (Roche cOmplete) in high‐performance liquid chromatography water (HPLC water), pH 8.0) and subjected to an N‐terminomics/TAILS workflow (Agbani et al. 2023; Anderson et al. 2020; Bhardwaj et al. 2024; Das et al. 2021; Das et al. 2023; Jagdeo et al. 2015; Jagdeo et al. 2018; Mallia‐Milanes et al. 2018; Wang et al. 2021; Ziegler et al. 2024). Samples were reduced with 5 mM DTT (Gold Biotechnology, St. Louis, MO) at 37°C for 1 h and alkylated with 15 mM IAA (GE Healthcare, Mississauga, ON) in the dark at room temperature for 30 min followed by quenching with 15 mM DTT. The pH was adjusted to 6.5 before the samples were isotopically labeled with a final concentration of 40 mM deuterated heavy formaldehyde (*CAPN2* ^*−/−*^) and with 40 mM light formaldehyde (WT) in the presence of 40 mM sodium cyanoborohydride overnight at 37°C. Next, samples were combined and were precipitated using acetone/methanol (8:1). The resulting pellet was resuspended in 1M NaOH and the proteins were subjected to trypsin (Promega, Madison, WI) digestion overnight at 37°C. For pre‐enrichment TAILS (pre‐TAILS)/shotgun proteomics, 10% of the trypsin digested samples were collected and the pH was adjusted to 3 with 100% formic acid. The rest of the samples were adjusted to a pH of 6.5 and incubated with a 3‐fold excess (w/w) of dendritic polyglycerol aldehyde polymer overnight at 37°C (Anowai et al. 2022; Das et al. 2023; Wang et al. 2021). Unbound peptides from the polymer‐bound peptides were filtered out by centrifugal filter unit with 10‐kDa cut‐off membrane (Amicon Ultra, Millipore) at 10,000*g* for 5 min. The flow‐through was collected and the Amicon columns were washed with 100 mM Tris–HCl, pH 6.5. The pH of the samples was adjusted to 3 with 100% formic acid. Both pre‐TAILS and TAILS samples were then desalted using Sep‐Pak C18 columns and lyophilized before submitting for LC–MS/MS analysis to the Southern Alberta Mass Spectrometry core facility, University of Calgary, Canada.

### 4.9 High‐performance liquid chromatography and mass spectrometry

All liquid chromatography and mass spectrometry experiments were carried out by the Southern Alberta Mass Spectrometry core facility at the University of Calgary, Canada. Analysis was performed on an Orbitrap Fusion Lumos Tribrid mass spectrometer (Thermo Fisher Scientific, Mississauga, ON) operated with Xcalibur (version 4.0.21.10) and coupled to a Thermo Scientific Easy‐nLC (nanoflow Liquid Chromatography) 1200 system. Tryptic peptides (2 μg) were loaded onto a C18 trap (75 μm 2 cm; Acclaim PepMap 100, P/N 164946; Thermo Fisher Scientific) at a flow rate of 2 μL/min of solvent A (0.1% formic acid and 3% acetonitrile in LC‐mass spectrometry grade water). Peptides were eluted using a 120 min gradient from 5 to 40% (5% to 28% in 105 min followed by an increase to 40% B in 15 min) of solvent B (0.1% formic acid in 80% LC‐mass spectrometry grade acetonitrile) at a flow rate of 0.3 μL/min and separated on a C18 analytical column (75 μm 50 cm; PepMap RSLC C18; P/N ES803; Thermo Fisher Scientific). Peptides were then electrosprayed using 2.3 kV into the ion transfer tube (300°C) of the Orbitrap Lumos operating in positive mode. The Orbitrap first performed a full mass spectrometry scan at a resolution of 120,000 FWHM to detect the precursor ion having a mass‐to‐charge ratio (m/z) between 375 and 1575 and a +2 to +4 charge. The Orbitrap AGC (Auto Gain Control) and the maximum injection time were set at 4 × 10^5^ and 50 ms, respectively. The Orbitrap was operated using the top speed mode with a 3 s cycle time for precursor selection. The most intense precursor ions presenting a peptidic isotopic profile and having an intensity threshold of at least 2 × 10^4^ were isolated using the quadrupole (isolation window of m/z 0.7) and fragmented with HCD (38% collision energy) in the ion routing Multipole. The fragment ions (MS2) were analyzed in the Orbitrap at a resolution of 15,000. The AGC, the maximum injection time, and the first mass were set at 1 × 10^5^, 105 ms, and 100 ms, respectively. Dynamic exclusion was enabled for 45 s to avoid the acquisition of the same precursor ion having a similar m/z (±10 ppm).

### 4.10 Proteomic data and bioinformatic analysis

Spectral data were matched to peptide sequences in the human UniProt protein database (version UP000005640 containing 83,385 entries and 20,644 genes) using the MaxQuant software package v.1.6.23, peptide‐spectrum match false discovery rate (FDR) of <0.01 for the shotgun proteomics data and <0.05 for the N‐terminomics/TAILS data. Search parameters included a mass tolerance of 20 ppm for the parent ion, 0.05 Da for the fragment ion, carbamidomethylation of cysteine residues (+57.021464), variable N‐terminal modification by acetylation (+42.010565 Da), and variable methionine oxidation (+15.994915 Da). For the N‐terminomics/TAILS data, the cleavage site specificity was set to semi‐ArgC (search for free N‐terminus) for the TAILS data and was set to ArgC for the preTAILS data, with up to two missed cleavages allowed. Significant outlier cut‐off values were determined after log(2) transformation by boxplot‐and‐whiskers analysis using the BoxPlotR tool (Spitzer et al. 2014). Database searches were limited to a maximal length of 40 residues per peptide. Peptide sequences matching reverse or contaminant entries were removed.

### 4.11 Reactome pathway analysis

To identify interconnectivity among proteins, the STRING‐db (Search Tool for the Retrieval of Interacting Genes) database was used to identify interconnectivity among proteins. The protein–protein interactions are encoded into networks in the STRING.v11 (Szklarczyk et al. 2019) database (https://string-db.org). Metascape (Zhou et al. 2019) (https://metascape.org) analysis was used to identify changes in functional enrichment, interactome analysis, and gene annotation. Our data were analyzed using *Homo sapiens* as our model organism at a false discovery rate of 1%.

### 4.12 Heatmaps of cleavage sites, TopFIND, and TopFINDer analysis

WebPICS was used using the website http://clipserve.clip.ubc.ca/pics. TopFIND (Lange and Overall 2011) and TopFINDer (Fortelny et al. 2015) analyses were performed using the website http://clipserve.clip.ubc.ca/topfind/. UniProt (https://www.uniprot.org/) and MEROPS (https://www.ebi.ac.uk/merops/index.shtml) were used to interpret the data.

### 4.13 Single‐cell RNA sequencing of acute myeloid leukemia dataset

The scRNA‐seq raw data for this study was obtained from GEO (Gene Expression Omnibus) with accession number “GSE241989.” Single‐cell RNA sequencing was performed on six bone marrow (BM) and six peripheral blood (PB) samples (see Table S7). Gene symbols were added to the GEO expression matrix using data from GPL18573 and GPL24676 platforms. We excluded low‐quality cells based on specific criteria: A minimum count of 500 unique genes and a maximum count of 30,000 were set. In addition, cells with more than 20% mitochondrial gene expression or fewer than 30 genes were discarded. Genes also were filtered by requiring expression in at least 20 cells, resulting in the removal of genes not expressed in sufficient numbers of cells. The data was then normalized and logarithmically transformed using the scanpy package (v1.10.1) with the functions scanpy.pp.normalize_per_cell (counts_per_cell_after = 1 × 10^4^) and scanpy.pp.log1p. A deep generative modeling technique called “scVI” was applied for batch effect correction and downstream analysis (Lopez et al. 2018).

Subsequently, we used the scanty.pp.highly_variable_genes function to identify highly variable genes. Using the function layer = “counts” and flavor = “seurat_v3,” 4000 top HVGs were selected based on their variance across cells. The nearest neighbors were computed using the sc.pp.neighbors function, leveraging the representation of cells generated by the scVI model. UMAP (Uniform Manifold Approximation and Projection) was used to visualize the high‐dimensional single‐cell data in a lower‐dimensional space. For automatic cell type annotation, we employed the celltypist package (v1.2.0) and utilized the celltypist.annotate function (model = “Immune_All_High.pkl,” majority_voting = True, min_prob = 3) (https://www.celltypist.org/models). This model, which encompasses detailed immune populations combined from 20 tissues of 18 studies (Version 2, published on 2022‐07‐16), was the most suitable model for annotating our dataset (Domínguez Conde et al. 2022).

### 4.14 Metabolomics

Metabolomics runs were performed as shown previously (de Almeida et al. 2022b; Michi et al. 2021) on a QExactive™ HF Hybrid Quadrupole‐Orbitrap™ Mass Spectrometer coupled to a Vanquish™ UHPLC System as a LC–MS analysis. Solvent A, 20 mM ammonium formate pH 3.0 in mass spectrometry grade H_2_O; Solvent B, mass spectrometry grade acetonitrile with 0.1% formic acid (% v/v). Chromatographic separation was attained using a binary solvent mixture of 20 mM ammonium formate at pH 3.0 in LC–MS grade water (Solvent A) and 0.1% formic acid (% v/v) in LC–MS grade acetonitrile (Solvent B) in conjunction with a 100 × 2.1 mm Syncronis™ HILIC LC column (Thermo Fisher Scientific) with a 2.1 μm particle size. The following gradients were used: 100% Solvent B (0–2 min), 100–80% Solvent B (2–7 min), 80–5% Solvent B (7–10 min), 5% Solvent B (10–12 min), 5–100% Solvent B (12–13 min), and 100% Solvent B (13–15 min). A sample injection volume of 2 μL was used. The mass spectrometer was run in negative full scan mode at a resolution of 240,000 scanning from 50 to 750 m/z. Metabolite analyses were completed using El Maven (v.0.12.0), a mass spectrometry data analysis software package (Clasquin et al. 2012). Metabolites were identified by matching observed m/z signals (±10 ppm) and chromatographic retention times to those observed from the reference Mass Spectrometry Metabolite Library. Data were analyzed and bioinformatic analyses were done using MetaboAnalystR 6.0 (Pang et al. 2024).

### 4.15 Statistical analysis

For the N‐terminomics analysis, an interquartile boxplot analysis was applied to determine the differential enrichment/upregulation of proteins (Spitzer et al. 2014). The FDR was generated by the bioinformatic tools used for pathway analysis. GraphPad Prism version 9 and Microsoft Excel were used for statistical analysis. The Student's *t* test and ANOVA determined significance between cells using the Prism software. A *p*‐value <0.05 was considered statistically significant.

## AUTHOR CONTRIBUTIONS

**Anjali Kapilan:** Writing – original draft; writing – review and editing; validation. **Mitchell Bulluss:** Writing – original draft; validation; writing – review and editing. **Alexander R. Ziegler:** Methodology; validation; visualization; writing – review and editing; formal analysis. **Mohamed Dabaja:** Formal analysis; writing – review and editing; methodology; validation; visualization. **Afshin Derakhshani:** Methodology; formal analysis; validation; writing – review and editing. **Anthonia Anowai:** Conceptualization; writing – original draft; formal analysis; methodology. **Victoria Armstrong:** Methodology; validation; formal analysis. **Rhiannon Campden:** Methodology; validation. **Daniel Young:** Methodology; validation; visualization; data curation. **Young Joo Sun:** Data curation; writing – review and editing. **Nichollas E. Scott:** Writing – review and editing; methodology; supervision. **Laura E. Edgington‐Mitchell:** Writing – review and editing; formal analysis; data curation; supervision; methodology. **Vinit B. Mahajan:** Methodology; writing – review and editing; data curation; supervision; funding acquisition. **Antoine Dufour:** Conceptualization; investigation; funding acquisition; writing – original draft; methodology; validation; visualization; writing – review and editing; supervision; data curation; formal analysis.

## Supporting information


**Data S1.** Supporting Information figures.

**Data S2.** Supporting Information tables.

**Table S7.** Supporting Information table.

## Data Availability

The data that support the findings of this study are openly available in PRIDE at https://www.ebi.ac.uk/pride/login, reference number PXD054339. Proteomics RAW data were deposited to ProteomeXchange via the Proteomics Identification Database (PRIDE) PXD054339 and under the login information: Username: reviewer_pxd054339@ebi.ac.uk, Password: HeKyZoj9u3Si. You can log in here: https://www.ebi.ac.uk/pride/login. It will be available upon publication. All data about identified and quantified peptides and proteins are included in Tables S1–S6.
