# Supplementary material for: N‐terminomics and proteomics analysis of Calpain‐2 reveal key proteolytic processing of metabolic and cell adhesion proteins
Source: Protein Sci. 2025 Apr 25;34(5):e70144. doi: 10.1002/pro.70144 (PMC12023407; doi:10.1002/pro.70144)
Supplement: Supplementary file 1 — Data S1. Supporting Information figures. [file PRO-34-e70144-s003.pdf]

Supplementary Figure 1

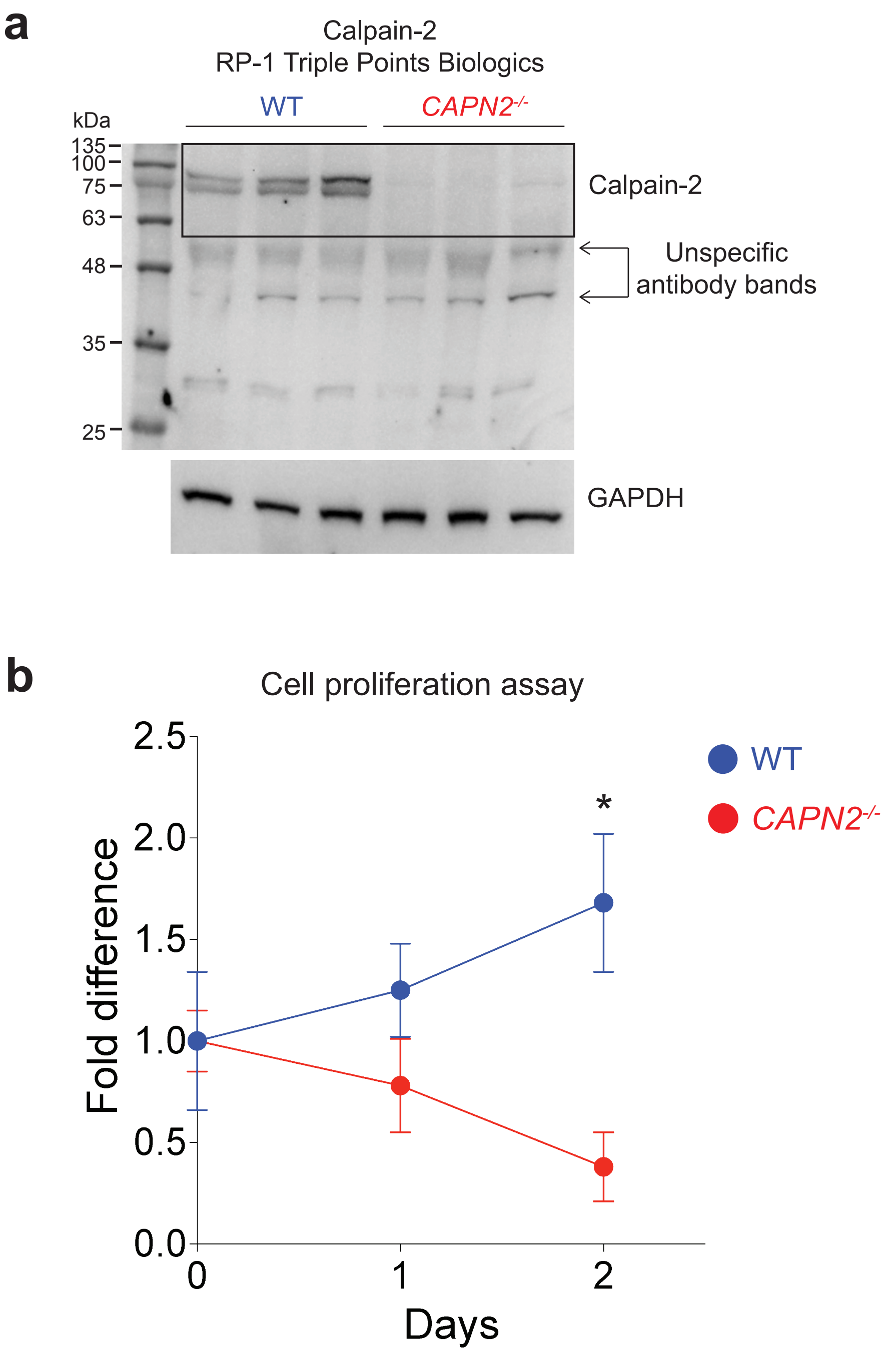

Supplementary Figure 1: Generation of WT and *CAPN2*$^{-/-}$ THP-1 cells and Western blot validation. **a)** Western blotting for Calpain-2 in WT and *CAPN2*$^{-/-}$ THP-1 cells using Calpain-2 antibody RP-1 from Triple Points Biologics. GAPDH was used as a loading control. **b)** Cell proliferation assay using WT and *CAPN2*$^{-/-}$ THP-1 cells. Student's t test *p<0.05.

Supplementary Figure 2

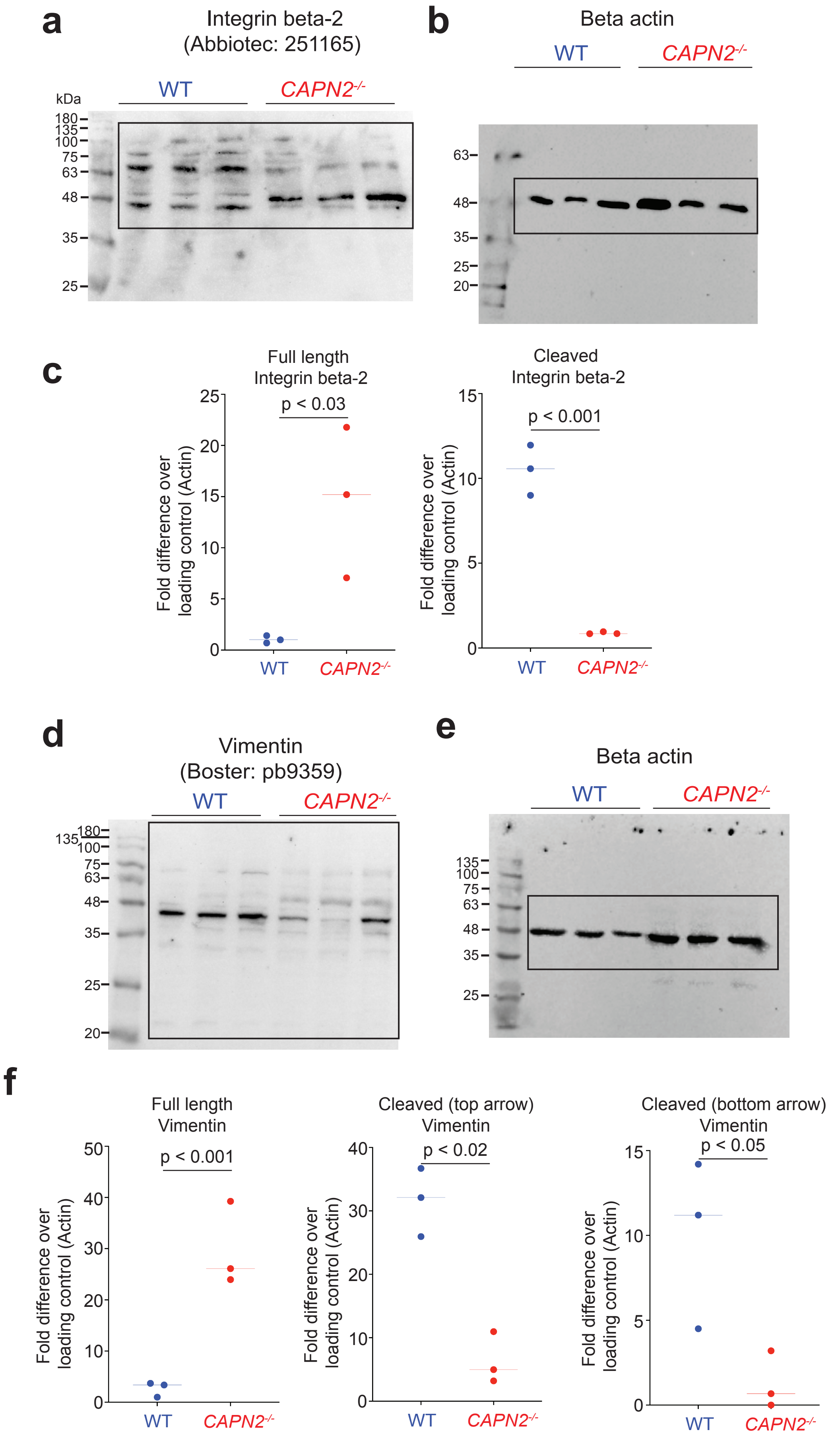

Supplementary Figure 2: WT and *CAPN2*$^{-/-}$ THP-1 cells and Western blot validation.
a) Western blotting for Integrin beta-2 and **b)** beta actin in WT and *CAPN2*$^{-/-}$ THP-1 cells. **c)** Quantification was done using ImageJ. Statistical analysis was performed using a Student's t-test from 3 independent samples. **d)** Western blotting for vimentin and **e)** beta actin in WT and *CAPN2*$^{-/-}$ THP-1 cells. **f)** Quantification was done using ImageJ. Statistical analysis was performed using a Student's t-test from 3 independent samples.

Supplementary Figure 3

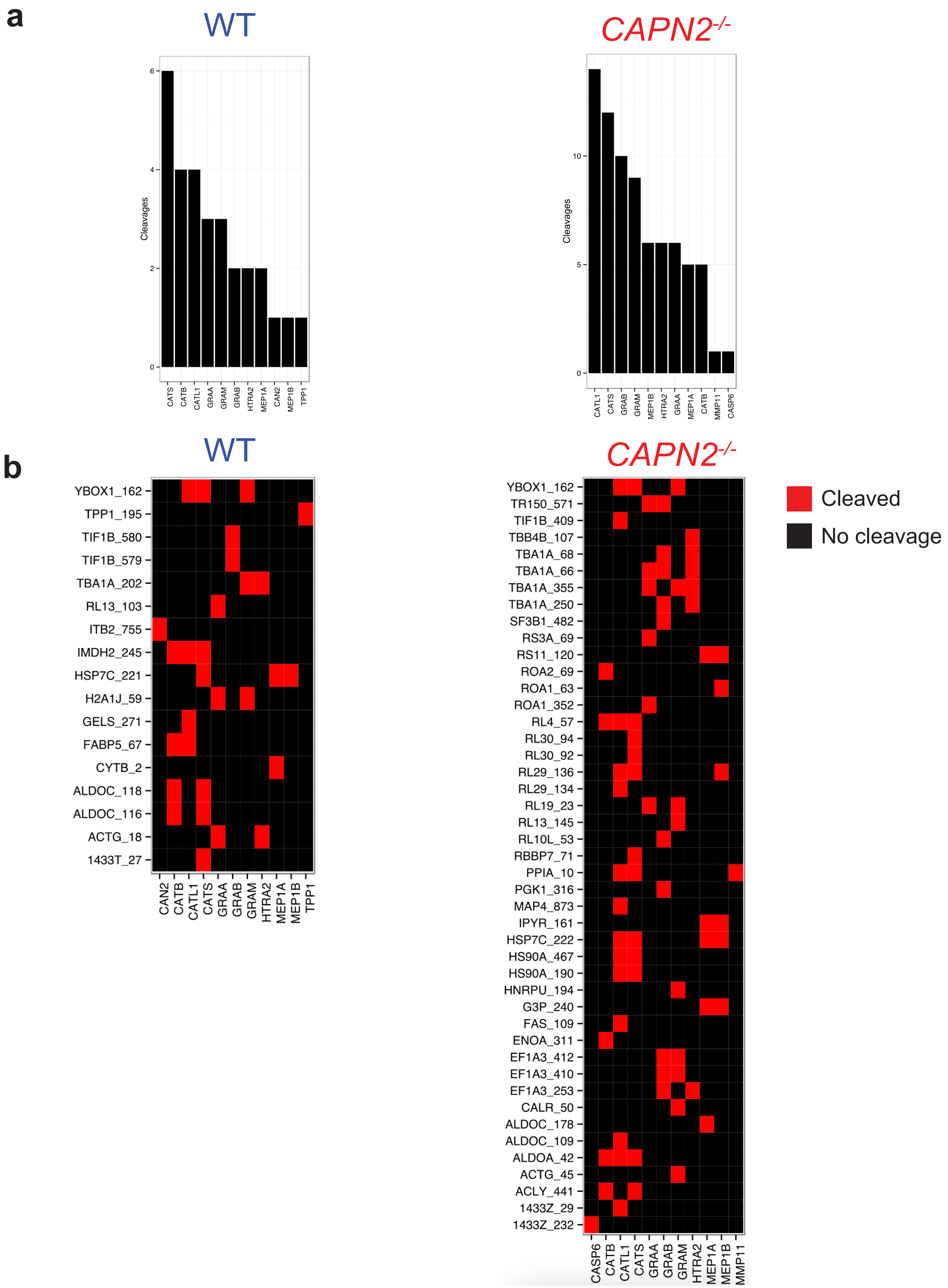

Supplementary Figure 3: TopFINDER and PathFINDER analysis of WT and *CAPN2*$^{-/-}$ THP-1 cells.
a) Number of known cleavages (y-axis) of various proteases (x-axis) as analyzed by TopFINDER and PathFINDER bioinformatic tools from *Left*, WT THP-1 cells and *Right*, *CAPN2*$^{-/-}$ THP-1 cells.
b) Known cleavages of substrates and amino acid positions (y-axis) by various proteases (x-axis) as analyzed by TopFINDER and PathFINDER bioinformatic tools from *Left*, WT THP-1 cells and *Right*, *CAPN2*$^{-/-}$ THP-1 cells. *Red*, cleaved substrates. *Black*, no cleavage.

Supplementary Figure 4

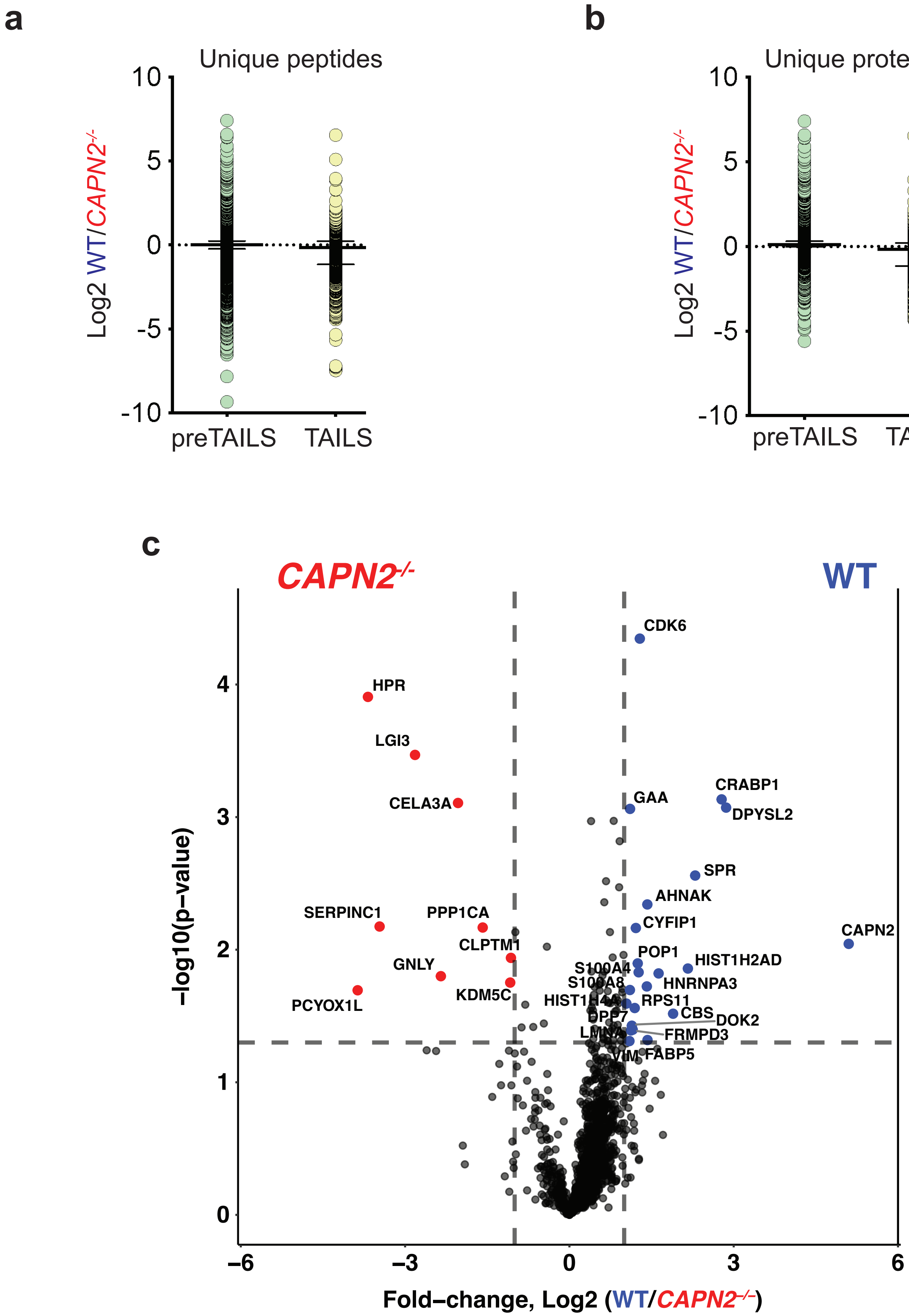

Supplementary Figure 4: **a)** Unique peptides identified in the preTAILS and TAILS analysis. **b)** Unique proteins identified in the preTAILS and TAILS analysis. **c)** Proteins from WT and *CAPN2*$^{-/-}$ THP-1 cells were analyzed by a two-way t test and visualized by volcano plot where significance is defined as intensities(log2(-/+PMA)) > 1 and -log10(p) > 1.3. The full list of peptides and proteins are shown in **Supplementary Tables 1-6**.
