# Supplementary material for: N‐terminomics and proteomics analysis of Calpain‐2 reveal key proteolytic processing of metabolic and cell adhesion proteins
Source: Protein Sci. 2025 Apr 25;34(5):e70144. doi: 10.1002/pro.70144 (PMC12023407; doi:10.1002/pro.70144)
Supplement: Supplementary file 3 — Table S7. Supporting Information table. [file PRO-34-e70144-s001.docx]

| **Sample annotation** | **GSM** | **Tissue** | **Disease** | **Sex** | **Genotype** |
| --- | --- | --- | --- | --- | --- |
| AML_PB_S1 | GSM7747225 | Peripheral blood | Primary acute myeloid leukemia cells | Male | Normal karyotype |
| AML_PB_S2 | GSM7747230 | Peripheral blood | Primary acute myeloid leukemia cells | Female | del(5q) |
| AML_PB_S3 | GSM7747231 | Peripheral blood | Primary acute myeloid leukemia cells | Male | Complex karyotype |
| AML_PB_S4 | GSM7747233 | Peripheral blood | Primary acute myeloid leukemia cells | Male | Complex karyotype |
| AML_PB_S5 | GSM7747234 | Peripheral blood | Primary acute myeloid leukemia cells | Female | Complex karyotype |
| AML_PB_S6 | GSM7747235 | Peripheral blood | Primary acute myeloid leukemia cells | Female | Normal karyotype |
| AML_BM_S7 | GSM7747223 | Bone marrow | Primary acute myeloid leukemia cells | Male | del(5q) |
| AML_BM_S8 | GSM7747224 | Bone marrow | Primary acute myeloid leukemia cells | Female | KMT2A-rearranged |
| AML_BM_S9 | GSM7747226 | Bone marrow | Primary acute myeloid leukemia cells | Female | Normal karyotype |
| AML_BM_S10 | GSM7747227 | Bone marrow | Primary acute myeloid leukemia cells | Male | KMT2A-rearranged |
| AML_BM_S11 | GSM7747228 | Bone marrow | Primary acute myeloid leukemia cells | Male | KMT2A-rearranged |
| AML_BM_S12 | GSM7747229 | Bone marrow | Primary acute myeloid  leukemia cells | Male | Normal karyotype |

**Table supplementary 7:** Patients’ characteristics data (obtained from GSE241989)

AML: Acute Myeloid Leukemia, PB: Peripheral blood, BM: Bone marrow
